# Clinical and socioeconomic impact of different types and subtypes of seasonal influenza viruses in children during influenza seasons 2007/2008 and 2008/2009

**DOI:** 10.1186/1471-2334-11-271

**Published:** 2011-10-12

**Authors:** Susanna Esposito, Claudio Giuseppe Molteni, Cristina Daleno, Antonia Valzano, Emilio Fossali, Liviana Da Dalt, Valerio Cecinati, Eugenia Bruzzese, Raffaella Giacchino, Carlo Giaquinto, Angie Lackenby, Nicola Principi

**Affiliations:** 1 Department of Maternal and Pediatric Sciences, Università degli Studi di Milano, Fondazione IRCCS Ca' Granda Ospedale Maggiore Policlinico, Milan, Italy; 2 Pediatric Department, University of Padua, Padua, Italy; 3 Department of Biomedicine of Evolutive Age, University of Bari, Bari, Italy; 4 Pediatric Department, Federico II University, Naples, Italy; 5 Infectious Disease Unit, IRCCS Ospedale Giannina Gaslini, Genoa, Italy; 6 Health Protection Agency, London, UK

**Keywords:** A/H1N1 influenza virus, children, influenza, pediatrics, viral types, viral subtypes

## Abstract

**Background:**

There are few and debated data regarding possible differences in the clinical presentations of influenza A/H1N1, A/H3N2 and B viruses in children. This study evaluates the clinical presentation and socio-economic impact of laboratory-confirmed influenza A/H1N1, A/H3N2 or B infection in children attending an Emergency Room because of influenza-like illness.

**Methods:**

Among the 4,726 children involved, 662 had influenza A (143 A/H1N1 and 519 A/H3N2) and 239 influenza B infection detected by means of real-time polymerase chain reaction. Upon enrolment, systematic recordings were made of the patients' demographic characteristics and medical history using standardised written questionnaires. The medical history of the children was re-evaluated 5-7 days after enrolment and until the resolution of their illness by means of interviews and a clinical examination by trained investigators using standardised questionnaires. During this evaluation, information was also obtained regarding illnesses and related morbidity among households.

**Results:**

Children infected with influenza A/H1N1 were significantly younger (mean age, 2.3 yrs) than children infected with influenza A/H3N2 (mean age, 4.7 yrs; p < 0.05)) or with influenza B (mean age, 5.2 yrs; p < 0.05). Adjusted for age and sex, children with influenza A/H3N2 in comparison with those infected by either A/H1N1 or with B influenza virus were more frequently affected by fever (p < 0.05) and lower respiratory tract involvement (p < 0.05), showed a worse clinical outcome (p < 0.05), required greater drug use (p < 0.05), and suffered a worse socio-economic impact (p < 0.05). Adjusted for age and sex, children with influenza B in comparison with those infected by A/H1N1 influenza virus had significantly higher hospitalization rates (p < 0.05), the households with a disease similar to that of the infected child (p < 0.05) and the need for additional household medical visits (p < 0.05).

**Conclusions:**

Disease due to influenza A/H3N2 viral subtype is significantly more severe than that due to influenza A/H1N1 subtype and influenza B virus, which indicates that the characteristics of the different viral types and subtypes should be adequately considered by health authorities when planning preventive and therapeutic measures.

## Background

A number of recent surveys of the total burden of pediatric seasonal influenza [1-4] have demonstrated that, although generally more dangerous in children at risk of influenza-related complications because of chronic underlying disease, it is also very common in otherwise healthy children, a considerable number of whom experience severe disease leading to excess hospitalisation rates, increased outpatient visits and antibiotic prescriptions, and (although rarely) even death [5]. Moreover, as children are particularly responsible for the spread of influenza, their disease has substantial socio-economic consequences because of the high rate of transmission among family members [3,6].

Among the influenza viruses causing disease in humans, types A and B are the most frequently isolated [7]. Despite all of them can infect subjects of all ages, community-based surveillance systems have found that A/H3N2 influenza virus is more frequently isolated in adults (including elderlies), influenza A/H1N1in young children and influenza B in school-age children [8]. However, there are few and debated data regarding possible differences in the clinical presentations of influenza A and B viruses in children. Some studies found a similar constellation of clinical signs and symptoms [9,10], whereas others indicate that influenza B infection may be more frequently associated with encephalitis, fatal pneumonia and myositis [11-13]. On the other hand, an ill appearance and more severe signs and symptoms of disease leading to higher hospitalisation rates are considered to be more common in children infected with influenza A [14-16].

Among the influenza A viruses, the H1N1 and H3N2 subtypes are the most common causes of disease [7,9], but little is known about their relative importance in determining the clinical features of the disease in pediatrics. Because the use of extensive vaccination against influenza among healthy children is not recommended in most industrialised countries [17,18], more data are needed to define whether the different subtypes may be risk factors for severe disease and hospitalisation. Furthermore, an awareness of the different clinical manifestations relating to viral subtype is particularly necessary when antiviral agents are used because the resistance of viral strains seems to be different [19,20].

This paper describes the clinical presentation and socio-economic impact of laboratory-confirmed influenza A (H1N1 and H3N2) or B infection in 901 otherwise healthy children attending an Emergency Room (ER) because of influenza-like illness during the influenza seasons 2007-2008 and 2008-2009.

## Methods

### Study design

This multicentre prospective study was carried out during the winters of 2007-2008 and 2008-2009 (from 1 November to 31 March) in the ERs of five children's hospitals in Italy (Milan, Padua, Genoa, Naples and Bari). The study protocol was approved by the Institutional Review Board of each participating centre; the written informed consent of a parent or legal guardian was required, and the older children were asked for their assent.

### Study population

The study enrolled subjects aged less than 15 years and without any underlying severe chronic disease who attended an ER because of an influenza-like illness as defined by the Italian Ministry of Health (http://www.ministerosalute.it) [21,22]. According to this definition, influenza-like disease was considered as an acute respiratory disease: of sudden onset; with fever (a temperature of > 38°C); accompanied by at least one of the following general symptoms: headache, generalised malaise, a feverish sensation (sweating and chills), asthenia; accompanied by at least one of the following respiratory symptoms: cough, pharyngodynia, nasal congestion.

The exclusion criteria were chronic diseases increasing the risk of complications of viral respiratory infections, including premature birth; chronic disorders of the pulmonary or cardiovascular systems including asthma; chronic metabolic diseases, including diabetes mellitus; neoplasia; kidney or liver dysfunction; hemoglobinopathies; immunosuppression; diseases requiring long-term aspirin therapy; and genetic or neurological disorders. Moreover, also those children whose parents could not assure the follow-up according to the study design were excluded. Because the study protocol did not include any invasive procedure and due to its epidemiologic nature, there was almost complete participation of the parents in the study.

During winter 2007-2008, the patients were enrolled two days per week (Wednesday and Sunday); during winter 2008-2009, they were enrolled every day. Upon enrolment in both seasons, systematic recordings were made of the patients' demographic characteristics and medical history using standardised written questionnaires [21,22]. The questions covered the signs and symptoms of the acute disease, laboratory and/or radiological examinations, prescribed drug therapy, the previous administration of influenza vaccine, family size and the number of siblings, the parents' education and occupation, the family's living conditions, and information concerning child care attendance. After a complete clinical examination, the study patients were classified into disease groups on the basis of signs and/or symptoms using well-established criteria [23]. In the case of signs and symptoms of more than one disease, the children were entered in the more severe disease group. Acute otitis media was diagnosed using pneumatic otoscopy [23] and community-acquired pneumonia by means of chest radiography [23].

A nasopharyngeal sample was collected from all of the children using a pernasal flocked swab, which was stored in a tube containing 1 mL of UTM-RT (Kit Cat. No. 360c, Copan Italia, Brescia, Italy).

The medical history of the children was re-evaluated 5-7 days after enrolment and every two days until the resolution of their illness by means of interviews and a clinical examination (in the outpatient clinic if they were discharged or in the ward if they were hospitalized) by trained investigators using standardised questionnaires [11,12]. During this evaluation, information was also obtained regarding illnesses and related morbidity among households. The patients' parents or legal guardians were asked to answer a list of questions regarding the outcome of the disease in their children and the involvement of other family members. All of the data of the study children and their households were verified from medical records.

### Laboratory assays

Each sample underwent real-time polymerase chain reaction (PCR) in order to identify A and B influenza viruses as previously described [24,25]. Viral RNA for subtyping was extracted from 150 μL of swab transport medium using a QIAxtractor, a VX reagent kit and a virus protocol (Qiagen, Crawley, UK). Reverse transcription plates were then set up using a Qiagility robot (Qiagen): 40 μL reactions were performed using 200 U MMLV reverse transcriptase (Invitrogen, Paisley, UK), 16 U rRNas in RNase inhibitor (Promega, Southampton, UK), 0.2 μg random hexamers (Thermo Scientific, West Sussex, UK), 0.4 mM of each dNTP, 7.5 mM MgCl_2_, and 1x Taq DNA Polymerase PCR Buffer (Invitrogen). The plates were incubated at room temperature for 10 minutes, then at 37°C for 45 minutes, and finally at 95°C for five minutes. Multiplex real-time PCR was performed using a Taqman Fast Universal PCR Master Mix (Applied Biosystems, Warrington, UK), fast cycling conditions, and the following primer-probes on an ABI 7500 Fast instrument (Applied Biosystems) (final concentrations in parentheses): A/H1-forward GGAATAGCCCCCCTACAATTG (1 μM); A/H1-reverse AATTCGCATTCTGGGTTTCCTA (1 μM); A/H1 probe NED-CGTTGCCGGATGGA-MGBNFQ (0.05 μM); A/H3-forward CCTTTTTGTTGAACGCAGCAA (1 μM); A/H3-reverse CGGATGAGGCAACTAGTGACCTA (1 μM); A/H3-probe VIC-CCTACAGCAACTGTTACC-MGBNFQ (0.25 μM); B-forward TCACGAAAAATACGGTGGATTAAA (0.75 μM); B-reverse TTTGGTTCCATTGGCMAGCT (0.75 μM); B-probe 6FAM-CCAATATGGGTGAAAAC-MGBNFQ (0.3 μM). The influenza A (including subtypes) and B RNA was relatively quantified; the criterion for a positive reaction was a cycle threshold (CT) of < 40 cycles.

### Statistical analysis

The data were analysed using SAS for Windows v. 9.1 (SAS Institute, Cary, NC, USA), and comparisons were made between the different viral types and subtypes. The continuous variables are presented as mean values ± standard deviation (SD) or median values with range, and the categorical variables as numbers and percentages. The continuous data were analysed using a two-sided Student's t-test after checking their normal distribution (based on the Shapiro-Wilk statistic) or a two-sided Wilcoxon's rank-sum test otherwise. The categorical data were analysed using contingency table analysis with the chi-squared or Fisher's test as appropriate. The multivariate odds ratios (OR) of the influenza virus subtypes (influenza A/H1N1, influenza A/H3N2, or influenza B), and the corresponding 95% confidence intervals (CI) were calculated using unconditional multiple logistic regression models, including terms for age and gender.

## Results

The study involved a total of 4,726 children (2,599 males; mean age ± SD, 3.34 ± 3.06 years): 1,170 (24.8%) during winter 2007-2008 and 3,556 (75.2%) during winter 2008-2009. Influenza A viruses were detected in 729 (15.4%): 164/1,170 (14.0%) in the first year and 565/3,556 (15.9%) in the second; influenza B viruses were detected in 239 (5.1%), 179/1,170 (15.3%) in 2007-2008 and 60/3,556 (1.7%; p < 0.0001) in 2008-2009. Despite the differences in prevalence during the two seasons, all the viral types co-circulated during 2007-2008 and 2008-2009, and no co-infection between different influenza types was observed. The influenza A viruses were subtyped in 150 of the 164 samples collected in the first year (91.5%) and 512 of the 565 samples collected in the second year (90.6%): subtype A/H1N1 was identified in respectively 126/150 (84%) and 17/512 cases (3.3%; p < 0.0001), and subtype A/H3N2 in 24/150 (16%) and 495/512 (96.7%; p < 0.0001). There was no between-centre difference in the prevalence of the different viral types or subtypes. As a preliminary evaluation showed that the demographic characteristics, clinical presentations, clinical outcomes and socio-economic impact were similar in the two seasons, the patients were considered together on the basis of the detected viral type and subtype. Consequently, the analysis was based on 662 children with influenza A (143 A/H1N1 and 519 A/H3N2) and 239 with influenza B.

Table 1 shows the demographic and clinical data of the enrolled children by the infecting influenza viral type and subtype. In comparison with the children with the A/H3N2 subtype, those with the A/H1N1 subtype were significantly younger (p < 0.05), experienced fever less frequently (including high grade fever) (p < 0.05), and were significantly less frequently affected by lower respiratory tract infections (p < 0.05) and fever without source (p < 0.05) upon enrolment. Wheezing and pneumonia were significantly more frequent among the patients infected with the A/H3N2 subtype (p < 0.05). With the exception of mean age as well as age distribution (p < 0.05) and the prevalence of acute bronchitis (p < 0.05) and myositis (p < 0.05), the demographic and clinical data of the patients with the A/H1N1 subtype were similar to those of the influenza B-positive children.

**Table 1:** Demographic and clinical presentation of the study patients by viral type and subtype.

Data	Viral findings		
	
	Influenza A/H1N1 (n = 143)	Influenza A/H3N2 (n = 519)	Influenza B (n = 239)
Males	79 (55.2)	312 (60.1)	125 (52.3)
Mean age ± SD, yrs	2.33 ± 1.40*°	4.69 ± 3.07	5.17 ± 3.21
Age groups, No. (%)			
< 2 yrs	39 (27.3)*	22 (4.2)	76 (31.8)*
2-5 yrs	103 (72.0)*°	491 (94.6)	74 (30.9)*
> 5 yrs	1 (0.7)°	6 (1.2)	89 (37.2)*
Presence of fever" (%)	109 (76.2)*	452 (87.1)	179 (74.8)*
High-grade fever° (%)	106 (74.1)*	416 (80.2)	169 (70.7)*
Respiratory tract infection (%)	128 (89.5)	436 (84.0)	205 (85.8)
Upper respiratory tract Infection (%)	107 (61.5)*	261 (50.3)	164 (68.7)*
Common cold (%)	69 (48.3)*	56 (10.8)	92 (38.5)*
Pharyngitis (%)	33 (23.1)	140 (26.9)	54 (22.5)
Acute otitis media (%)	9 (6.3)	46 (8.9)	14 (5.9)
Croup (%)	4 (2.8)	11 (2.1)	4 (1.7)
Lower respiratory tract infection (%)	21 (14.6)*	175 (33.7)	41 (17.2)*
Acute bronchitis (%)	24 (16.8)°	83 (15.9)	21 (8.8)*
Wheezing (%)	3 (2.0)*	58 (11.1)	7 (2.9)*
Pneumonia (%)	0 (0.0)*	34 (6.5)	13 (5.4)
Gastroenteritis (%)	5 (3.5)	17 (3.3)	11 (4.6)
Fever without source (%)	6 (4.2)*	52 (10.0)	7 (2.9)*
Febrile seizures (%)	4 (2.7)	14 (2.7)	4 (1.7)
Myositis (%)	0 (0.0)°	0 (0.0)	12 (5.0)

"Defined as an axillary temperature of ≥37.6°C or a rectal temperature of ≥38°C; °defined as an axillary temperature of ≥39°C or a rectal temperature of ≥39.5°C. SD, standard deviation. °p < 0.05 *vs *influenza B; *p < 0.05 *vs *A/H3N2; no other statistically significant differences.

Table 2 summarises clinical outcomes and drug use. In comparison with the children infected by the A/H3N2 subtype, those infected by A/H1N1 had a significantly lower hospitalisation rate (p < 0.05), a significantly shorter duration of hospitalisation (p < 0.05) and absence from school (p < 0.05), and made significantly less frequent use of antibiotics (p < 0.05), antipyretics (p < 0.05), aerosol therapy (p < 0.05) and steroids (p < 0.05). However, although the hospitalisation rate was significantly lower among the children infected by the A/H1N1 subtype than in those infected by influenza B (p < 0.05), the clinical outcomes and drug use were similar in these two groups.

**Table 2:** Clinical outcome and drug use among the study population by viral type and subtype.

Data	Viral findings		
	
	Influenza A/H1N1 (n = 143)	Influenza A/H3N2 (n = 519)	Influenza B (n = 239)
Clinical outcome			
Hospitalisation rate, No.(%)	6 (4.2)*°	87 (16.8)	30 (12.5)
Duration of hospitalisation, mean days ± SD	5.2 ± 3.4*	7.6 ± 4.4	4.6 ± 2.6*
Absence from school, mean days ± SD	6.10 ± 4.93*	7.61 ± 4.44	6.43 ± 5.01*
Drug use, No. (%)			
Antibiotics (%)	109 (76.2)*	470 (90.5)	179 (74.9)*
Antipyretics (%)	113 (79.0)*	469 (90.3)	190 (79.5)*
Aerosol therapy (%)	36 (25.2)*	222 (42.8)	63 (26.3)*
Steroids (%)	6 (4.2)*	57 (10.9)	8 (3.3)*

SD, standard deviation. °p < 0.05 *vs *influenza B; *p < 0.05 *vs *A/H3N2; no other statistically significant differences.

Table 3 shows the socio-economic impact of the influenza viral types and subtypes on the household contacts of the study children. The household impact of the A/H1N1 subtype was quite different from that of the A/H3N2 subtype. The prevalence of disease similar to that of the infected children (p < 0.05), the number of medical visits (p < 0.05), the number of antibiotic prescriptions (p < 0.05), the number of working days lost by mothers (p < 0.05) and fathers (p < 0.05), and the number of school days lost by siblings (p < 0.05) were significantly lower in the case of the A/H1N1 subtype. The socio-economic impact of the A/H1N1 subtype was also significantly lower than that due to influenza B virus for the majority of the studied variables (p < 0.05).

**Table 3:** Socioeconomic impact of influenza viruses on household contacts of the study children by viral type and subtype.

	Viral findings		
	
Data	Households of Influenza A/H1N1-positive children (n = 363)	Households of Influenza A/H3N2-positive children (n = 1,296)	Households ofB-positive children (n = 597)
Disease similar to that of the infected child (%)	70/363 (19.3)*°	417/1,296 (32.2)	159/597 (26.6)*
Mothers, No. (%)	16/143 (11.2)*°	173/519 (33.3)	58/239 (24.3)*
Fathers, No. (%)	18/139 (12.9)*	103/519 (19.8)	37/239 (15.5)
Siblings, No. (%)	36/81 (44.4)*°	141/258 (54.7)	64/119 (53.8)
Additional medical visits (%)	45/363 (12.4)*°	325/1,296 (25.1)	125/597 (20.9)*
Mothers, No. (%)	12/143 (8.4)*°	134/519 (25.8)	46/239 (19.2)*
Fathers, No. (%)	6/139 (4.3)*°	55/519 (10.6)	21/239 (8.8)
Siblings, No. (%)	27/81 (33.3)*°	136/258 (52.8)	58/119 (48.7)
Antibiotic prescriptions (%)	16/363 (4.4)*	131/1,296 (10.1)	42/597 (7.0)
Mothers, No. (%)	3/143 (2.1)*	38/519 (7.3)	11/239 (4.6)
Fathers, No. (%)	1/139 (0.7)*	29/519 (5.6)	5/239 (2.1)
Siblings, No. (%)	12/81 (14.8)*°	64/258 (24.8)	26/119 (21.8)
Lost working days by mothers, mean ± SD	4.33 ± 2.16*	6.01 ± 2.57	3.46 ± 2.51*
Lost working days by fathers, mean ± SD	1.39 ± 1.52*	3.36 ± 2.12	2.03 ± 2.57*
Lost school days by siblings, mean ± SD	3.16 ± 4.01*	4.93 ± 3.91	3.39 ± 3.15*

SD, standard deviation. ^*p *< 0.05 *vs *influenza B; °p < 0.05 *vs *influenza B; *p < 0.05 *vs *A/H3N2; no other statistically significant differences. Lost parental working days were due to caring for ill children and their personal illness.

Table 4 shows the multivariate ORs of the influenza virus subtypes and their 95% CIs on the basis of the characteristics of the study patients and their households. All the variables mentioned in the preceding Tables were evaluated, but only those who showed significant differences between different set of viruses upon adjustment for age and sex are reported. Adjusted for age and sex, children with influenza A/H3N2 in comparison with either A/H1N1 or with B virus infection showed significantly more often fever (including high-grade fever) (p < 0.05), lower respiratory tract involvement (p < 0.05), fever without source (p < 0.05), need of hospitalization (p < 0.05), absence from school (p < 0.05), requirement of antibiotics (p < 0.05) and antipyretics (p < 0.05) as well as aerosol therapy (p < 0.05) and steroids (p < 0.05), the households with a disease similar to that of the infected child (p < 0.05), the need for additional household medical visits (p < 0.05) and antibiotic prescriptions (p < 0.05), a longer period of lost work days by parents (p < 0.05) and school days by siblings (p < 0.05). Adjusted for age and sex, children with influenza B in comparison with those infected by A/H1N1 influenza virus had significantly higher hospitalization rates (p < 0.05), the households with a disease similar to that of the infected child (p < 0.05) and the need for additional household medical visits (p < 0.05).

**Table 4:** Significant odds ratios (OR) and 95% confidence intervals (CI) of the associations between the influenza subtypes and the characteristics of the study patients and their households.

	Influenza A/H1N1 vs influenza A/H3N2 OR (95% CI)	Influenza A/H1N1 vs influenza B OR (95% CI)	Influenza B vs influenza A/H3N2 OR (95% CI)
Presence of fever			
No	1 (reference)	1 (reference)	1 (reference)
Yes	0.68 (0.41-0.99)	1.03 (0.66-1.79)	0.65 (0.39-0.94)
Presence of high-grade fever"			
No	1 (reference)	1 (reference)	1 (reference)
Yes	0.76 (0.55-0.97)	0.98 (0.62-1.55)	0.75 (0.53-0.96)
Upper respiratory tract infection			
No	1 (reference)	1 (reference)	1 (reference)
Yes	1.34 (1.10-1.81)	0.86 (0.53-1.36)	1.31 (1.08-1.79)
Lower respiratory tract infection			
No	1 (reference)	1 (reference)	1 (reference)
Yes	0.60 (0.39-0.84)	1.19 (0.68-1.91)	0.69 (0.43-0.93)
Fever without source			
No	1 (reference)	1 (reference)	1 (reference)
Yes	0.64 (0.43-0.85)	1.12 (0.66-1.93)	0.61 (0.40-0.87)
Hospitalisation			
No	1 (reference)	1 (reference)	1 (reference)
Yes	0.57 (0.37-0.82)	0.61 (0.40-0.88)	0.84 (0.53-1.36)
Duration of hospitalisation (days)			
< 7 days	1 (reference)	1 (reference)	1 (reference)
≥7 days	0.62 (0.40-0.90)	1.11 (0.85-1.42)	0.60 (0.39-0.85)
Absences from school (days)			
< 7 days	1 (reference)	1 (reference)	1 (reference)
≥7 days	0.64 (0.43-0.91)	0.96 (0.61-1.74)	0.62 (0.42-0.98)
Antibiotic use			
No	1 (reference)	1 (reference)	1 (reference)
Yes	0.65 (0.44-0.93)	1.14 (0.66-1.98)	0.68 (0.47-0.94)
Antipyretic use			
No	1 (reference)	1 (reference)	1 (reference)
Yes	0.69 (0.40-0.91)	0.93 (0.54-1.91)	0.67 (0.39-0.96)
Aerosol therapy			
No	1 (reference)	1 (reference)	1 (reference)
Yes	0.65 (0.43-0.88)	1.18 (0.67-1.89)	0.62 (0.36-0.93)
Steroids			
No	1 (reference)	1 (reference)	1 (reference)
Yes	0.69 (0.39-0.94)	0.93 (0.58-1.67)	0.70 (0.49-0.95)
Household diseases similar to that of the infected child			
No	1 (reference)	1 (reference)	1 (reference)
Yes	0.63 (0.41-0.92)	0.71 (0.42-0.94)	0.78 (0.50-0.98)
Additional medical visits among households			
No	1 (reference)	1 (reference)	1 (reference)
Yes	0.68 (0.40-0.94)	0.74 (0.46-0.97)	0.79 (0.55-0.99)
Household antibiotic prescriptions			
No	1 (reference)	1 (reference)	1 (reference)
Yes	0.65 (0.42-0.93)	0.87 (0.62-1.25)	0.91 (0.68-1.29)
Lost working days by mothers			
< 3 days	1 (reference)	1 (reference)	1 (reference)
≥3 days	0.65 (0.34-0.89)	1.13 (0.54-1.92)	0.69 (0.38-0.96)
Lost working days by fathers			
< 3 days	1 (reference)	1 (reference)	1 (reference)
≥3 days	0.58 (0.36-0.85)	0.87 (0.62-1.35)	0.65 (0.41-0.93)
Lost school days by siblings			
< 3 days	1 (reference)	1 (reference)	1 (reference)
≥3 days	0.76 (0.54-0.97)	0.92 (0.70-1.38)	0.77 (0.58-0.98)

Odds ratios from multivariate logistic models, including terms for age and gender.

## Discussion

Most of the data collected during this study confirm that influenza can cause substantial clinical and socio-economic problems not only in children at risk of complications but also in otherwise healthy children [1-5]. Our study population consisted only of otherwise healthy children, the great majority of whom attended the ERs with fever and respiratory tract infection, and a considerable number were hospitalised and received drug therapy, thus confirming that influenza leads to high rates of hospitalisation and antipyretic and antibiotic prescriptions also among healthy pediatric subjects [1,2].

Data showing that more than 70% of patients with influenza received antibiotics are impressive in the light of campaigns to reduce antibiotic usage in Europe, especially related to viral infections like influenza. The use of rapid tests to confirm influenza diagnosis during epidemic periods in settings like ERs may be helpful for reducing this inappropriate antibiotic abuse [26].

The findings also confirm that influenza can present with fever without other accompanying symptoms, and that its initial presentation may be gastroenteritis or febrile seizures. Moreover, as previously reported [13], we found that influenza B infection can present with unusual manifestation, including myositis. However, the prevalence of acute otitis media (AOM) was significantly lower than that reported in other studies of the complications of influenza [15,27] but similar to that previously found by our group [6,26]. These differences can be explained by the fact that we analysed signs and symptoms upon enrolment, whereas AOM can arise some days after the beginning of the disease. Moreover, the antibiotic prescription rate was very high in our population, thus reducing the probability of AOM due to bacterial superinfection. Finally, our findings confirm that influenza has a significant impact on households and considerable socio-economic consequences among parents and siblings [3,6].

The new study findings are those related to the impact of the different viral types and subtypes, which show that disease due to influenza A/H3N2 virus is significantly more severe than that due to influenza A/H1N1 viral subtype and influenza B virus upon adjustment for age and gender. In studies showing the greater clinical importance of influenza A virus [18-20], this finding has been mainly attributed to the fact that younger children are at higher risk of severe influenza disease and that influenza A infection is usually more common among patients in the first five years of life. Our data suggest that the viral subtypes themselves may play different roles in conditioning the clinical and socio-economic impact of influenza infection in children. Although our patients infected by the A/H1N1 subtype were significantly younger than those infected by the A/H3N2 subtype, the severity of the disease was less and quite similar to that due to influenza B virus. Moreover, a specific multivariate analysis upon adjustment for age group and gender has been done showing that the greater severity of A/H3N2 in this and other studies [28-31] may be due to specific characteristics leading to greater virulence.

This study was mainly focused on different types and subtypes of seasonal influenza viruses, and no other respiratory virus was evaluated. Further studies that include possible differences and co-infections with other respiratory viruses should be useful also for a better understanding of influenza viruses' pathogenesis. Moreover, only the ER setting was evaluated and findings in primary care could be partially different. Finally, our data refer to the influenza seasons 2007-2008 and 2008-2009, before the circulation of the pandemic A/H1N1 influenza virus. It is therefore possible that these conclusions may not apply to the present situation in which the new A/H1N1 subtype has completely replaced its predecessor. However, to the best of our knowledge this is the largest study that evaluates clinical as well as socioeconomic characteristics of different types and subtypes of seasonal influenza viruses in pediatrics.

## Conclusions

Disease due to influenza A/H3N2 virus is significantly more severe than that due to influenza A/H1N1 viral subtype and influenza B virus, which indicates that the characteristics of the different viral types and subtypes should be adequately considered by health authorities when planning preventive and therapeutic measures.

## List of abbreviations

(AOM): Acute otitis media; (CI): confidence interval; (CT): cycle threshold; (ER): Emergency Room; (OR): odds ratio; (PCR): polymerase chain reaction; (SD): standard deviation.

## Competing interests

The authors declare that they have no competing interests.

## Authors' contributions

SE and NP designed the study and co-wrote the manuscript; CGM performed the statistical analysis and together with CD and AV carried out the real-time PCR; EF, LDD, VC, EB, RG, and CG visited the patients and collected the swabs; AL supported in the laboratory assays. All authors read and approved the final manuscript.

## Pre-publication history

The pre-publication history for this paper can be accessed here:

http://www.biomedcentral.com/1471-2334/11/271/prepub
